# Diagnosing glaucoma in primary eye care and the role of Artificial Intelligence applications for reducing the prevalence of undetected glaucoma in Australia

**DOI:** 10.1038/s41433-024-03026-z

**Published:** 2024-03-21

**Authors:** Catherine Jan, Mingguang He, Algis Vingrys, Zhuoting Zhu, Randall S. Stafford

**Affiliations:** 1 Centre for Eye Research Australia, Royal Victorian Eye and Ear Hospital, East Melbourne, VIC Australia; 2 Ophthalmology, Department of Surgery, Faculty of Medicine, Dentistry & Health Sciences, University of Melbourne, Melbourne, VIC Australia; 3 Lost Child’s Vision Project, Sydney, NSW Australia; 4 Centre for Eye and Vision Research, The Hong Kong Polytechnic University, Kowloon, TU428 Hong Kong SAR; 5 Department of Optometry and Vision Sciences, The University of Melbourne, Melbourne, VIC Australia; 6 Stanford Prevention Research Center, Stanford University School of Medicine, Stanford, CA USA

**Keywords:** Physical examination, Epidemiology

## Abstract

Glaucoma is the commonest cause of irreversible blindness worldwide, with over 70% of people affected remaining undiagnosed. Early detection is crucial for halting progressive visual impairment in glaucoma patients, as there is no cure available. This narrative review aims to: identify reasons for the significant under-diagnosis of glaucoma globally, particularly in Australia, elucidate the role of primary healthcare in glaucoma diagnosis using Australian healthcare as an example, and discuss how recent advances in artificial intelligence (AI) can be implemented to improve diagnostic outcomes. Glaucoma is a prevalent disease in ageing populations and can have improved visual outcomes through appropriate treatment, making it essential for general medical practice. In countries such as Australia, New Zealand, Canada, USA, and the UK, optometrists serve as the gatekeepers for primary eye care, and glaucoma detection often falls on their shoulders. However, there is significant variation in the capacity for glaucoma diagnosis among eye professionals. Automation with Artificial Intelligence (AI) analysis of optic nerve photos can help optometrists identify high-risk changes and mitigate the challenges of image interpretation rapidly and consistently. Despite its potential, there are significant barriers and challenges to address before AI can be deployed in primary healthcare settings, including external validation, high quality real-world implementation, protection of privacy and cybersecurity, and medico-legal implications. Overall, the incorporation of AI technology in primary healthcare has the potential to reduce the global prevalence of undiagnosed glaucoma cases by improving diagnostic accuracy and efficiency.

## Abstract (zh)

青光眼是全球最常见的不可逆失明性疾病, 超过70%的患者尚未确诊。目前尚无治愈方法。对于青光眼患者来说, 早期诊断对于阻止视力恶化至关重要, 本综述旨在: 找出全球范围内, 特别是澳大利亚普遍存在的青光眼患病率严重被低估的原因；以澳大利亚的医疗保健为例阐明初级医疗保健在青光眼诊断中的作用；讨论如何利用最新的人工智能 (AI) 技术来提高诊断的准确性。青光眼在人口老龄化的国家普遍存在, 通过适当的治疗可以改善视力结局, 因此对于医疗实践至关重要。在澳大利亚、新西兰、加拿大、美国和英国等国家, 验光师是初级眼科保健的守护者, 青光眼检测通常由他们负责。然而, 眼科专业人员在青光眼诊断能力上存在显著差异。利用AI对眼底彩色照片的视神经进行自动化识别技术可帮助验光师快速、高诊断效能地识别高风险变化, 并降低图像解读方面的挑战。尽管具有潜力, 但在将AI应用于初级保健场景之前, 还需要解决一些重要的屏障和挑战, 包括外部验证、高质量的实际应用、保护隐私和网络安全以及医疗法律问题。总之, 将AI纳入初级保健中将通过提高诊断准确性和效率来减少未确诊的青光眼病例的全球发病率。

## Introduction

Glaucoma poses a significant public health challenge. Despite its impact, a large percentage of glaucoma cases remain undetected, especially in primary eye care settings [1]. The primary objective of our review article is to explore the reasons behind the underdiagnosis of glaucoma, particularly in Australia, and discuss how recent advances in Artificial Intelligence (AI) applications can be utilised to improve diagnostic outcomes.

The review article will delve into the global significance of glaucoma and undetected cases, highlighting the prevalence of underdiagnosis in different regions, including Australia. We will examine the challenges faced by primary healthcare providers in accurately diagnosing glaucoma, such as the complexity of the diagnostic process and the lack of specialised training and equipment. Furthermore, we will discuss the potential of AI applications in addressing these challenges and improving glaucoma detection rates.

Our article will provide a comprehensive overview of the clinical detection of glaucoma, focusing on the use of digital imaging technologies, such as monoscopic fundus photos and optical coherence tomography (OCT), as well as the importance of visual field testing. We will also highlight the importance and challenges of glaucoma care at the primary eye care level, emphasising the roles of general practitioners (GPs) and optometrists in glaucoma diagnosis and management.

The section dedicated to AI applications will explore the potential of AI algorithms in glaucoma detection. We will present the performance of AI algorithms using fundus photography, OCT, and visual fields, showcasing their accuracy in diagnosing glaucoma. Additionally, we will discuss the deployment of AI products in clinical practice, addressing potential risks and the need for validation studies and protocols to ensure the reliability and safety of AI-assisted diagnosis.

## Global significance of glaucoma and undetected glaucoma

The global significance of glaucoma and its underdiagnosis is a significant public health issue due to its status as the leading cause of irreversible blindness worldwide [2–4]. Glaucoma is the second leading cause of blindness among people aged 55 and over in Australia [5]. Despite its impact, over 70% of people with glaucoma remain undiagnosed globally [1]: 78%−94% for Africa [6], 72%−84% for Asia [7, 8], 57%−68% for Europe [9], 62%−78% for North America [10], 75%−88% for Latin America [11] and, 50%−60% for Australia and Oceania [7, 12–14]. The estimated numbers of glaucoma cases worldwide in 2020 are 52.7 million detected versus 43.8 million undetected. These numbers will increase in 2040 to 79.8 million and 67.1 million, respectively [1, 15].

The organisation of eye care services, skill levels of involved health care providers, and access to relevant health technologies contribute to glaucoma underdiagnosis. Organisational issues, particularly who provides eye care and who pays for it, varies globally. In Australia, the U.S., and many western European nations, routine eyecare is often fragmented between optometrists and primary healthcare physicians (GP), while ophthalmologists handle specialised patients and issues on referral from each. Inadequate diagnostic technology and provider training further exacerbate underdiagnosis. A new form of technology, Artificial Intelligence (AI), offers a potentially favourable transformation for glaucoma diagnosis. This promise, however, will depend on key financial, logistical, and organisational hurdles. The Australia example of diagnosing and managing glaucoma could be globally useful in addressing the significant public health issue of glaucoma underdiagnosis.

A non-systematic literature review was conducted based on articles published in peer-reviewed journals from the following databases: PubMed, oScopus, Medline-OVID, EMBASE, Cochrane Library, Web of Science and nongovernment organisation reports on 15 April 2023 using search terms to identify articles with no limitations on the publication year. The search combinations included “Glaucoma Care”, “Glaucoma Management”, “Glaucoma Diagnosis”, “Glaucoma Progression”, “Artificial Intelligence”, “Deep Learning”, “Machine Learning”, “Neural Networks”, “Bayesian Networks”, “Clinical Tools”, and “Primary Care”.

## Overview of clinical detection of glaucoma

The hallmark of glaucoma is ganglion cell neurodegeneration in the optic nerve that is typically associated with functional loss. Consequently, diagnosis is largely based on optic nerve head (ONH) assessment and visual field (VF) testing, supplemented by several auxiliary tests and patient history (Table 1). Since the disorder is irreversible and has no available cure, early detection is vital for halting progressive visual impairment in glaucoma patients [16].

**Table 1:** Equipment required by optometrist in diagnosis, assessment, and management of glaucoma, as specified by the Optometrist Board of Australia.

Assessment	Equipment required^a^
Optic nerve head and RNFL	Slit lamp and fundus lens; fundus photography and/or OCT of posterior pole
Threshold visual fields	Automated threshold perimetry tailored to the patient and degree of visual field loss
Anterior chamber angle	Slit lamp and gonioprism
Intraocular pressure	Goldmann applanation tonometer
Central corneal thickness	Pachymeter, anterior OCT or slit-lamp

^a^Clinical Practice Guide for the Diagnosis and Management of Open Angle Glaucoma 2020. (Accessed 31 Jan 2023).

### Detecting structural defects in glaucoma

Digital imaging technologies have become useful not only for documenting ONH and retinal nerve fibre layer (RNFL) changes (glaucomatous optic neuropathy or GON), but also for providing an objective, quantitative and convenient method to assist clinicians in glaucoma diagnosis.

#### Monoscopic fundus photos

Accurate and consistent detection of the optic disc and RNFL due to progressive retinal ganglion cell death is the key to glaucoma diagnosis [17]. Traditionally, mydriasis is recommended to obtain a stereoscopic view for optic disc assessment. But a recent study shows that monoscopic optic disc photography provides non-inferior diagnostic accuracy for the clinical evaluation of all optic disc characteristics and glaucoma likelihood [18]. Monoscopic disc images offer a fast and affordable method to aid in GON detection.

#### Optical coherence tomography (OCT)

OCT is a non-contact, optical imaging technique that uses low coherence interferometry to measure backscatter from different layers of the optic nerve head and retina [19]. In a few seconds, it captures structural information such as RNFL thickness, ganglion cell layer thickness, disc size, and minimum rim width [20, 21] (the minimum rim width identifies the nerve fibre thickness passing out of the eye back to the brain). Pragmatically, longitudinal data are unlikely to be available to establish the initial glaucoma diagnosis. Therefore, structural information from a single observation (cross-sectional observation) could be considered as standard for diagnosis [20].

### Detecting functional impairment in GON

#### Visual field testing

Visual sensitivity progressively declines in glaucoma. Standard automated perimetry (SAP) over the central 24°−30° of the visual field is the current recommended procedure for visual field testing in glaucoma [19, 22]. It measures retinal sensitivity to increments of light at many locations across the visual field. The 24−2 grid (central 24 indicates that the central 24 degrees of visual field and the next number indicates how the grid of points is aligned to the visual axis) is the preferred test strategy for this purpose, although the 30−2 is sometimes used to rule out other neurological conditions [23]. Repetition of visual field testing at baseline using the same threshold strategy is essential to establish a baseline so that the earliest presence of glaucomatous functional defect can be identified, and to accommodate for learning effects in performing the visual field test [24]. Visual field testing is recommended more than twice a year [25], with a minimum of three-times per year suggested to overcome test variability and identify progression in newly diagnosed glaucoma patients [26]. This frequency of testing will obviously challenge health care systems. Recent work indicates that a combination of structural and functional measures (VF and OCT) gives the greatest sensitivity for diagnosis and identifying progression in glaucoma [27].

## Importance and challenges of glaucoma care at primary levels in Australia

### Challenges faced by primary healthcare providers in glaucoma diagnosis

Glaucoma diagnosis is a complex process that requires targeted evaluation by eye care professionals. While primary healthcare providers play an important role in glaucoma screening, they face numerous challenges in accurate diagnosis. The diagnostic process includes history taking, assessment of the ONH and RNFL, measurement of intraocular pressure (IOP), evaluation of the anterior angle and anterior chamber by slit lamp to exclude angle closure or secondary causes of glaucoma, and VF evaluation. Although these assessments are essential for definitive glaucoma diagnosis, not all are required for screening to identify presumptive glaucoma (Box 1).

**Fig. 1:**
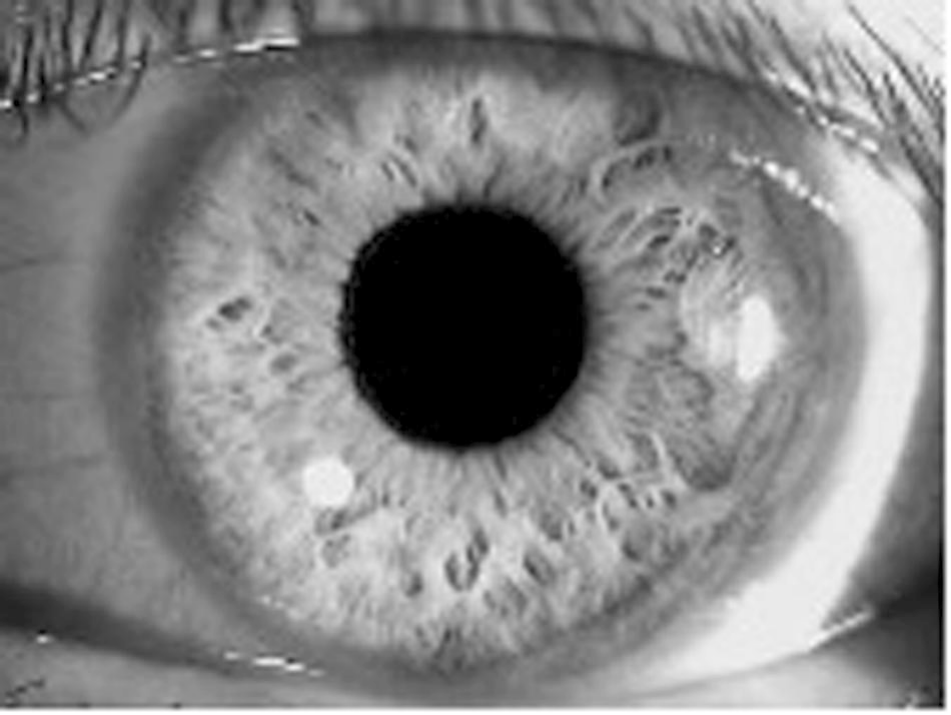
showing a wide-open anterior angle with flashlight examination, as evident by the small shadow (banana shaped on left) indicating a flat iris. The flashlight is located at the zygomatic arc on the right of the image.

**Fig. 2:**
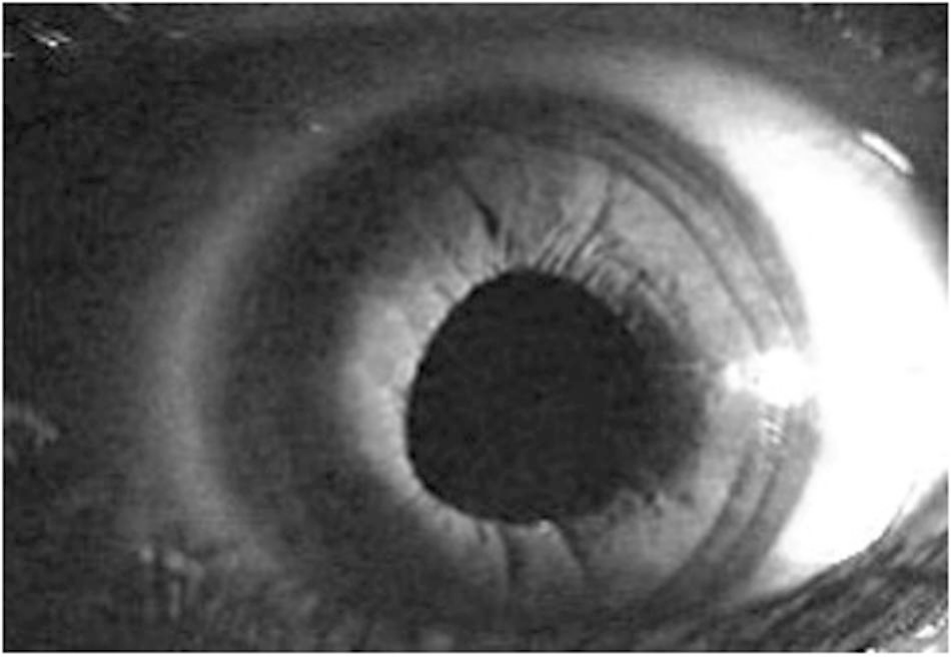
showing a closed anterior angle on flashlight examination. This indicates a prominent and forward placed iris protruding into the anterior chamber, causing angle closure. The flashlight is located at the zygomatic arc on the right of the image.

A study conducted in Victoria, Australia, found that out of 4744 individuals, 72 cases of referable glaucoma were identified, of whom 35 (49%) were undiagnosed [1]. The primary cause of misclassification was the lack of VF screening, as 97% of their 35 missed cases failed a VF screening test during a subsequent date. The study also found that 66% of the missed cases had an enlarged cup-to-disc ratio, which was not identified during their previous eyecare visits [1]. Perhaps this finding is not unexpected as cup-disc evaluation using ophthalmoscopy is challenging, and GPs are not versed in performing this procedure, so they may not identify abnormality from normal variations. However, in the case of this study, most participants who had enlarged cup-disc ratios had seen an optometrist or an ophthalmologist in the past year, and their physical state did not provide adequate evidence for further evaluation. This implies that cup-disc evaluation is a complex and challenging undertaking even for ophthalmic practitioners and would likely lead to high false positive or false negative outcomes if undertaken by GPs.

The implementation of AI has the potential to alleviate some of the challenges faced by primary healthcare providers in glaucoma diagnosis Box 2. AI technology can aid in the interpretation of images of the ONH, RNFL, and VF, providing a more accurate and consistent assessment. However, before AI can be widely adopted in primary healthcare settings, several issues must be resolved first, as we will discuss later in this article.

#### A case of primary eye care in glaucoma diagnosis in Australia

Australia’s healthcare system is founded on the principles of universal health care and a robust public insurance programme, ensuring that all citizens and permanent residents and New Zealand citizens have access to free and high-quality medical services [28]. This fundamental aspect has played a pivotal role in the successful implementation of national cancer screening programmes for bowel, cervical and breast cancer [29]. The achievements of these screening programmes underscore the capability of Australia’s healthcare system in effectively executing universal screening initiatives. Considering the accomplishments in cancer screening, there is a strong foundation for the feasibility of developing and executing a universal glaucoma screening programme. The existing infrastructure, along with the commitment to preventive healthcare, positions Australia favourably to address glaucoma detection and management comprehensively. By leveraging its healthcare system’s strengths and incorporating the latest advancements in medical technology, Australia has the potential to enhance the early diagnosis and treatment of glaucoma, ultimately safeguarding the vision and overall health of its population.

In Australia there are theoretically two models of primary care for glaucoma detection—by GPs and by optometrists. In practice, however, GPs are not trained to diagnose glaucoma and do not have access to testing equipment, therefore the burden of glaucoma diagnosis falls to optometrists. However, optometrists may face challenges in performing all the required procedures for glaucoma diagnosis (Table 1), especially when under time pressures, as experienced by all primary health care practitioners.

Starting in 2009, topical glaucoma medications prescribed by optometrists became available at subsidised prices (Pharmaceutical Benefits Scheme, PBS) [30]. In 2020, there were 6043 registered optometrists in Australia, with 65% having prescribing endorsement [31]. Despite being given increasing independence in prescribing [32], optometrists have not widely embraced this role. In 2015, latanoprost was the drug most prescribed by optometrists under PBS. However, while about 40% of these optometry prescriptions are for glaucoma management, they only accounted for 1.3% of all glaucoma prescriptions [32]. There were around 1000 full-time equivalent (FTE) ophthalmologists and 4800 FTE optometrists employed in Australia in 2019, or 4 FTE ophthalmologists and 19 FTE optometrists per 100,000 population [25]. Thus, optometrists were not highly active in managing glaucoma with most cases referred to ophthalmology.

#### Current glaucoma care guidelines for optometry

A patient who presents to an optometrist with glaucoma risk factors during a routine eye examination is recommended to undergo further clinical investigations to determine if glaucoma is present. For this purpose, the Optometry Board of Australia guidelines [33] recommend that practising optometrists have certain equipment to form a proper evaluation and differential diagnosis (Table 1). If this equipment is not available, or if testing cannot be undertaken to satisfy the requirements of Table 1, then a referral should be made to another optometrist or ophthalmologist for specialised testing and interpretation of the test results.

The glaucoma assessment needs to be made by all optometrists, whether endorsed for the use of scheduled medicines (therapeutically endorsed) or not. If an optometrist is therapeutically endorsed, they are authorised to diagnose and initiate glaucoma treatment independently. Once the diagnosis is established and the treatment started, patients are to be seen by an ophthalmologist within 4 months [33]. This referral reflects that surgical intervention is sometimes a viable first-choice treatment option and should be considered in all cases [34, 35].

Although this surgical related review produces a best-case option, delays in seeing an ophthalmologist can be substantial due to high caseloads [31]. A patient must be referred by a primary care provider (optometrist or GP) to gain access to specialist eye care in a public hospital in Australia and to be eligible for a rebate under the national health insurance act (Medicare Benefits Schedule) [36]. A 2020 study found that 72% of glaucoma referrals to the public hospital system came from optometrists [37]. However, the study also found that the median wait-time for patients to be seen by an ophthalmologist at the hospital can be as long as 400 days [37]. This delay in treatment can lead to irreversible sight loss, which can be avoided if optometrists/GPs prescribe IOP reducing drugs on diagnosis, given that most optometrists are therapeutically qualified. It will also act to minimise the possibility of vein occlusion associated with lengthy exposure to elevated IOP. Patients can suffer harm and sight loss due to 22 week delay in achieving hospital attendance [38], which supports an argument for therapeutic intervention on diagnosis to reduce the potential for sight loss prior to hospital attendance.

Box 1 List of clinical procedures recommended for glaucoma screening in primary healthcare settings [43], where a slit lamp is unavailable

- Family history, history on risk factors of glaucoma
- Shadow test using flashlight or ophthalmoscope at temple (Figs. 1, 2)
- Optic Nerve Head assessment in terms of cup-disc ratio
- Visual Field screening
- Age-specific reviews (40+) doing these tests for all patients for age 40 and above, and repeat every five years up to 60, and biannually after that age.

Box 2 A glossary of key terms **Artificial intelligence (AI)**. The study of agents that receive precepts from the environment and perform actions. It is concerned with both understanding and building intelligent entities—machines that can compute how to act effectively and safely in a wide variety of novel situations [94].

**Machine learning (ML)**. A subfield of artificial intelligence that is concerned with the study of machines that use algorithms to identify patterns in data [95].

**Supervised ML**. A type of machine learning task that aims at predicting the desired output (such as separating “glaucomatous” from “non-glaucomatous”). It involves the use of data labelled by clinical experts to train machines and develop statistical models.

**Unsupervised ML**. A type of machine learning task that aims at inferring underlying patterns in unlabelled data.

**Deep learning (DL)**. A subfield of machine learning that employs artificial neural networks with many layers to identify patterns in data [96].

### Why are so many people with glaucoma not diagnosed?

The cause of the high prevalence of undetected glaucoma is multifactorial [39–41]. Firstly, as above, glaucoma diagnosis requires the consideration of complex diagnostic tests, many of which are not available to GPs and some optometry settings. Additionally, glaucoma (except for *acute* angle closure glaucoma) is hard to diagnose as patients have few or no symptoms of the disease [4]. Visual symptoms are rare in early and middle stages of glaucoma, thus glaucoma is coined “the silent thief of vision”[1].

Secondly, while universal screening for glaucoma is not cost-effective in Western countries [42], detection of glaucoma relies on primary care providers, namely optometrists and GPs. However, GPs rarely test patients for glaucoma due to a lack of training and specialised equipment [43]. Fortunately in Australia, we have universal health coverage for routine comprehensive eye examinations in optometry settings, and all optometry students are trained in glaucoma care. However, variation exists in ONH assessment, which could have contributed to the high rate of undetected glaucoma reported in Australia [44].

**Fig. 3:**
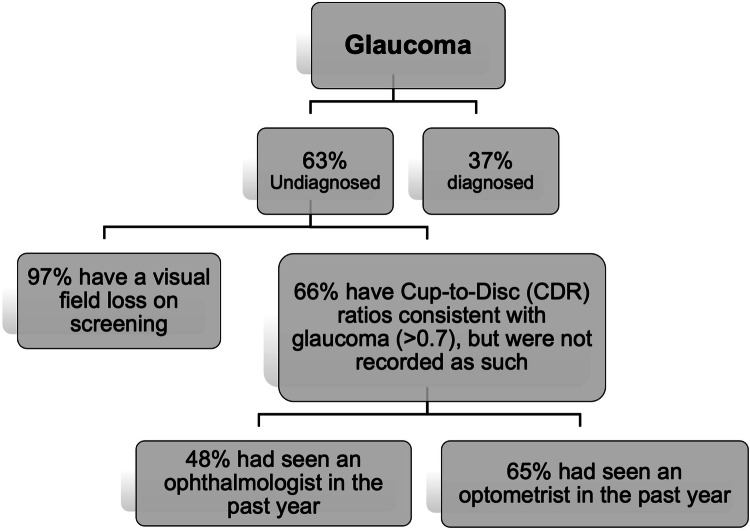
Undiagnosed glaucoma in Victoria, Australia (calculated on data from Wong et al. [1]).

A population study [1] in Victoria found that undiagnosed glaucoma was as high as 63% (Fig. 3). Of the undiagnosed cases, 66% had seen an optometrist in the past year, 97% did not have a visual field test performed, and 66% had Cup-to-Disc (CDR) ratios consistent with glaucoma (>0.7) but were not recorded as such. Of note, out of those undiagnosed cases with CDR > 0.7, 65% had seen an optometrist and 48% had seen an ophthalmologist in the past year [1]. This indicates that glaucomatous disc changes are challenging to detect, which may explain the significant missed diagnosis. The prevalence of undiagnosed disease is even higher in minority populations [45, 46].

Standardising the detection of GON based on structural and functional changes can help bridge the gap in glaucoma detection. AI can potentially play a useful role in this regard by providing consistent imaging interpretation and identifying high risk cases for closer consideration by the optometrist.

## Potential of AI (artificial intelligence) in glaucoma diagnosis in primary eye care settings

### Context and definition

When Deep Blue (a chess-playing computer developed by IBM [47]) defeated Garry Kasparov (the youngest world chess champion in history) in 1997, the defenders of human supremacy moved humanity’s battleground to Go (an abstract strategy board game for two players in which the aim is to surround more territory than the opponent. The number of legal board positions in Go has been calculated to be approximately 2.1 × 10^170^ [48], which is far greater than the number of atoms in the observable universe, estimated to be of the order of 10^80^) [49]. Piet Hut, an astrophysicist and Go enthusiast, predicted that it would take “a hundred years before a computer beats humans at Go—maybe even longer.” But in just under 20 years since Deep Blue vs. Kasparov, a computer programme developed by neuroscientist and chess prodigy Demis Hassabis and his DeepMind team surpassed all human players at Go [50].

As in other domains, AI is rapidly changing the healthcare landscape. Recent advancements in AI have introduced promising opportunities for efficient and cost-effective glaucoma detection programmes. Many studies have shown that AI algorithms now equal or exceed expert diagnostic accuracy for many conditions, particularly when the diagnosis is based on image interpretation, such as in dermatology and radiology [51–57]. Eye care is the frontrunner of the AI revolution in health care because diagnosing eye conditions heavily depends on imaging. In 2018, IDx-DR (Digital Diagnostics), designed to detect diabetic retinopathy and diabetic macular oedema, became the first FDA-approved autonomous AI device in any field of medicine [58]. The integration of AI into the glaucoma diagnostic process has the potential to significantly reduce costs and resource burdens and provides a potential to yield more accurate diagnosis.

### Overview for performance of AI algorithm in glaucoma detection

#### AI using fundus photography

AI can help address the issue of variation in the assessment of ONH and RNFL changes. Segmentation and structured learning from various studies achieved an accuracy between 94% and 98% [59, 60] in reaching a correct diagnosis from fundus photos in glaucoma. Various deep learning algorithms based on fundus features such as the cup-to-disc ratio achieved area under receiver operating curve (AROC) between 0.53 and 0.996 in differentiating healthy from glaucomatous eyes [61–64], with a sensitivity ranging from 96% to 100% [61, 65], and specificity of 98% [64, 65]. Recently, Machine Learning (ML) algorithms developed from the interrogation of 50,000 fundus photos achieved an area under curve (AUC) of 0.986 with 95.6% sensitivity and 92% specificity for identifying referable GON [66]. AI-based tools can provide standardised and objective assessments, leading to more accurate and consistent diagnoses.

#### AI using OCT

The RNFL thickness is one common parameter utilised for glaucoma diagnosis [67] and became a key focus in ML using OCT images. Since 2005, studies have reported the performance of ML algorithms analysing OCT imaging data from peripapillary RNFL thickness maps and the macular ganglion cell complex for detecting GON, with AROC values ranging from 0.69 to 0.99 [68–75]. A recent study showed that Deep Learning (DL) network achieved an AROC of 0.94 for detecting GON using unsegmented OCT volumes of the optic nerve head [76]. Given the cost of OCT this application of AI is likely best retained for specialist or optometry practice where ocular OCT can be applied for many other purposes.

#### AI using visual fields

**Fig. 4:**
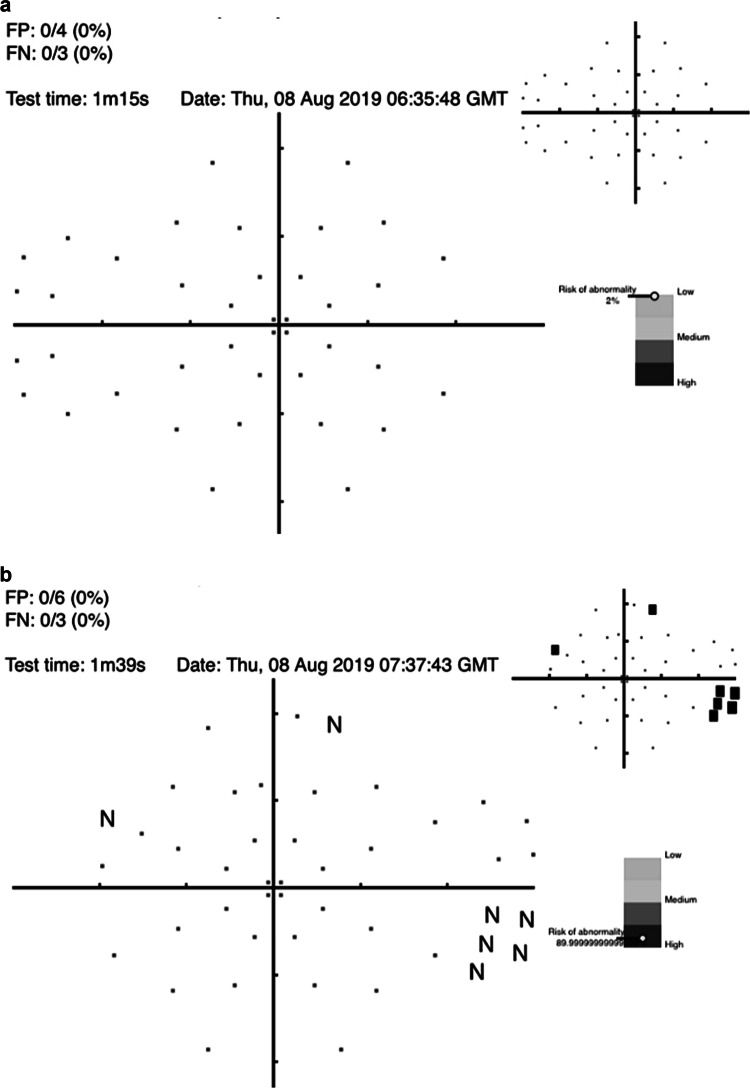
An example of visual field screening. **a** visual field screening result using the Melbourne Rapid Fields app [84] for a 57 year old male showing the risk for glaucoma is low. **b** visual field screening result using the Melbourne Rapid Fields app [84] for a 68 year old female showing the risk for glaucoma is high. The data were collected using Melbourne Rapid Fields app and required about 1−1.5 min for testing (from Chia et al. [93]).

AI algorithms to diagnose glaucoma using datasets derived from VF testing have been studied since 1994 [77–81]. Notably, DL algorithms to diagnose glaucoma with data from standard automated perimetry (SAP) with Humphrey VF 24-2 and 30-2 SITA standard VF test outperformed the diagnostic accuracy of glaucoma experts in differentiating normal from glaucomatous VFs, with a sensitivity of 93% and specificity of 83% [82]. Furthermore, algorithms trained using a combination of OCT images and SAP VF results reached an AROC of 0.98 for identifying patients with glaucoma [83]. The cost of many dedicated testing devices is prohibitive for general application but the recent advent of a cheap screening option (the Melbourne Rapid Fields app [84]) makes this suited for such purposes. Figure 4 illustrates examples for such screening options and where the interpretation of results is given in terms of a “probability of abnormality score” (coloured bar) to assist the clinician’s decision making process and identify high risk cases.

#### Multi-modal AI models

Research has also utilised multimodal structural data to enhance the assessment of glaucomatous structural damage from optic disc photographs for segmentation and detection [85, 86]. A recent multimodal model was developed using the Xception model for image feature extraction and various ML algorithms such as random forest (RF), support vector machine (SVM), dense neural network (DNN), and others showed impressive area under the receiver operating characteristic curve (AUROC) values for the different algorithms: RF had an AUROC of 99.56%, SVM had 99.59%, and DNN had 99.10% while analysing the vertical cup-to-disc ratio and mean RNFL thickness in the detection of glaucoma in a population with high incidence of myopia [87]. Another recent study showed that FusionNet based on bimodal input of VF and OCT paired data demonstrated superior performance to algorithms based on VF or OCT alone [88].

### Incorporation of AI products in Australian primary care

Recent advancements in imaging technologies (such as OCT and retinal photos) allow primary care clinicians and eye specialists to identify the structural damage caused by glaucoma [89]. However, these advances come with a high cost of equipment and significant time required to interpret the image or results. Moreover, it is becoming increasingly challenging for busy primary eyecare clinics to undertake such imaging without incurring substantial costs.

Furthermore, there is substantial variation in the visual interpretation of ONH features among clinicians [90]. This variation can be present between different clinicians (inter-observer variation) and between assessments made at different times by the same clinician (intra-observer inter-session variation). A recent study [90] involving 197 ophthalmic clinicians from 22 countries showed substantial under diagnosis based on optic nerve head photos from patients with known glaucoma. Ophthalmology trainees (22%) and comprehensive ophthalmologists (24%) consistently underestimated the likelihood of glaucoma. This level of underestimation contributes to undetected cases of glaucoma, supporting the need for AI-assisted clinical evaluation.

**Fig. 5:**
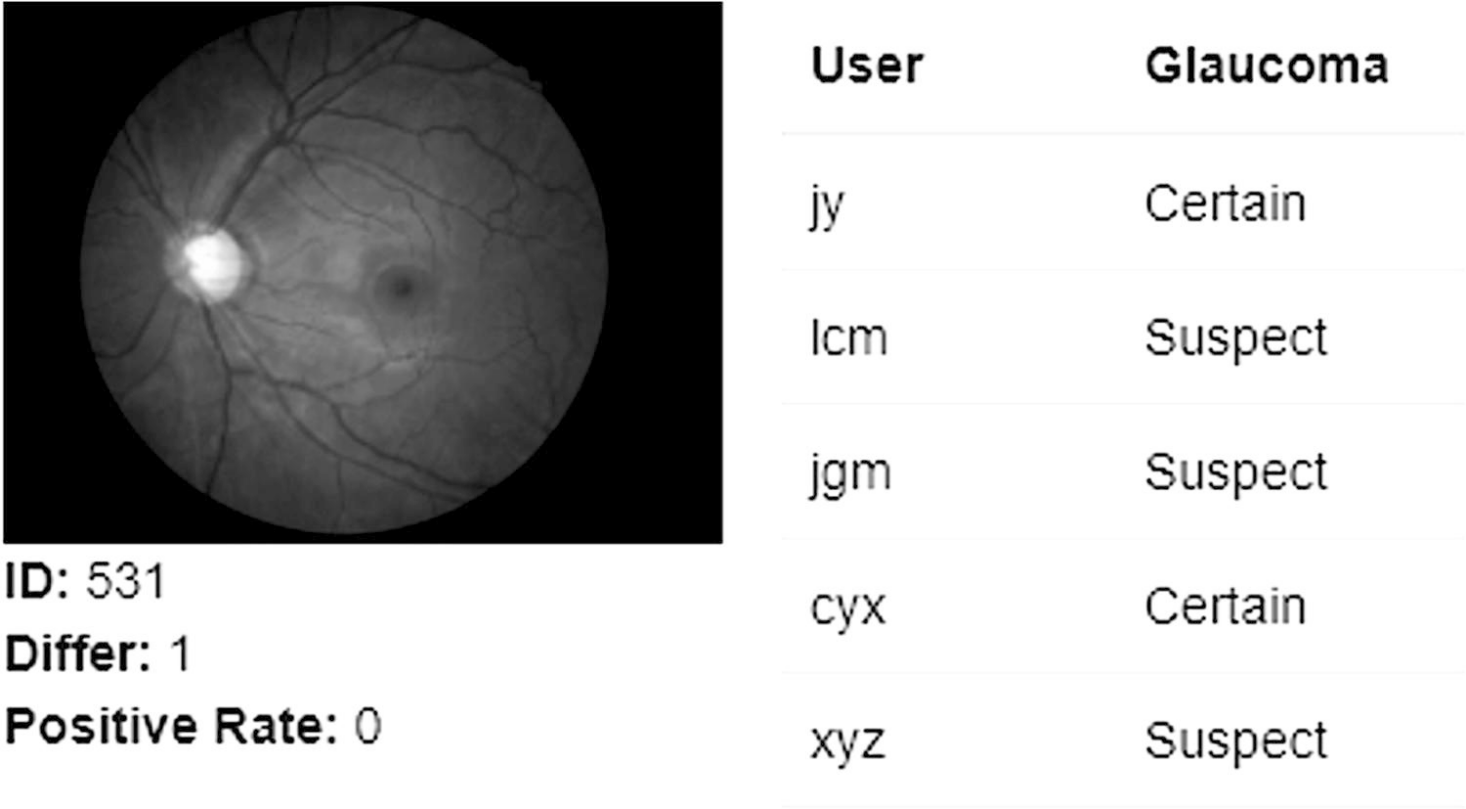
Image with grading results that showed disagreement even among glaucoma specialists, but AI produced consistent results. Each user represents one glaucoma specialist (internal data from Prof. Mingguang He, obtained in March, 2022).

Automation by AI offers an opportunity to mitigate the time and cost challenges of image analysis and reduce diagnostic variation compared to human clinicians (Fig. 5). Figure 5 shows the outcome of an optic nerve image processed with AI that was consistently graded as having glaucoma by AI but returned variable diagnoses between 5 expert clinicians.

**Fig. 6:**
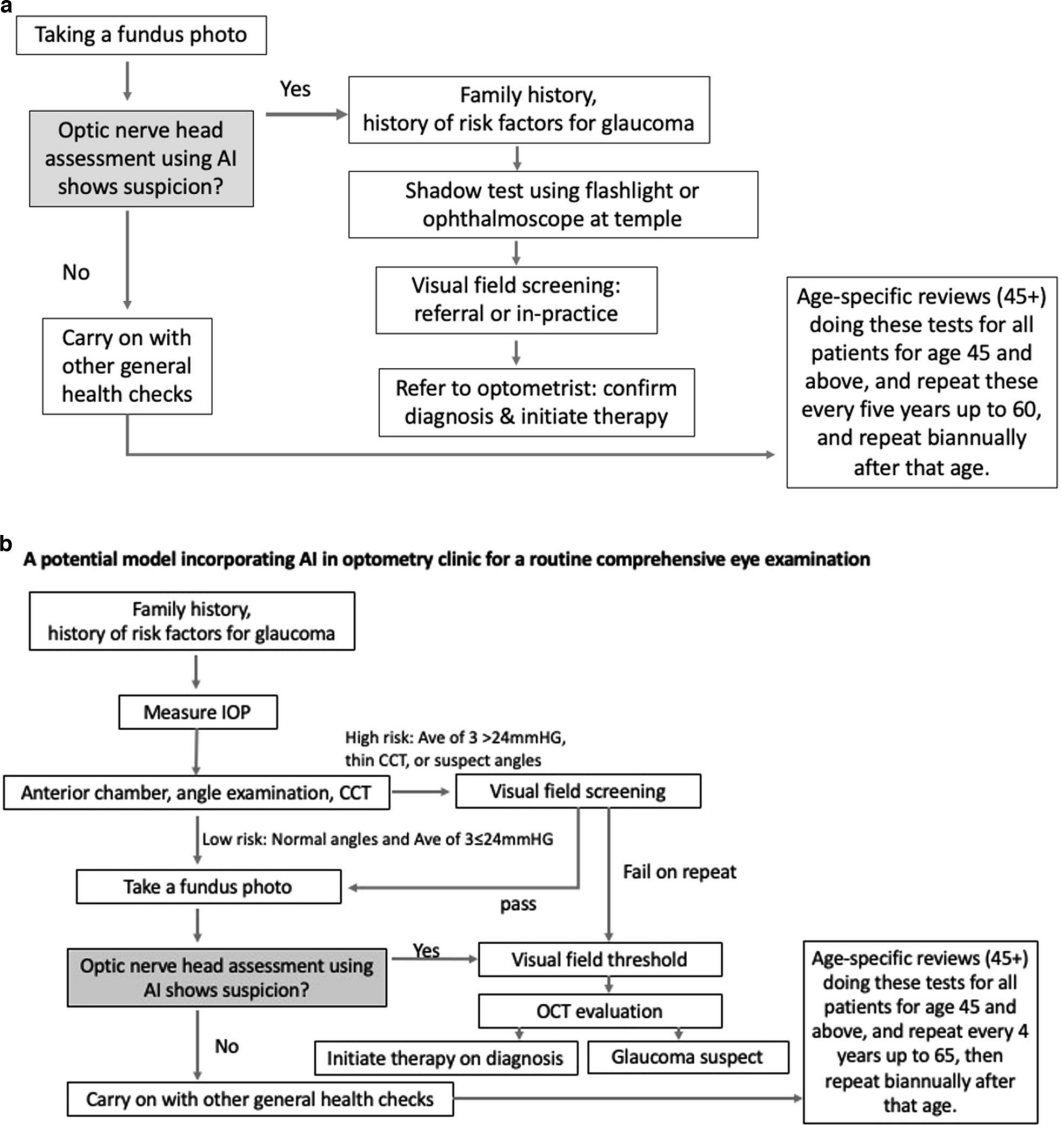
AI models in primary eye care. **a** A potential model incorporating AI in GP clinics for a routine health examination which achieves rapid therapeutic intervention (unpublished protocols). **b** A potential model incorporating AI in optometry clinics for a routine comprehensive eye examination (unpublished protocols).

Figure 6a, b propose two potential models where AI can be incorporated into primary care settings for glaucoma detection. The assortment of tests was developed based on currently available guidelines for glaucoma detection in primary care settings [43, 91] and adopting easy to use and interpret methods for diagnosis. More research is required to investigate the best AI-assisted model for the detection of glaucoma in primary care settings.

### Managing potential risks of AI use in glaucoma diagnosis

Significant progress in ML and imaging technologies enable AI to identify glaucoma signs. However, despite rapid progress in AI for glaucoma detection under laboratory settings, its real-world application has not been fully realised. The lack of a unified “gold standard” for glaucoma diagnosis is perhaps one of the biggest challenges that AI faces, as different AI algorithms focus on different aspects of the disease. For instance, the AI that focuses on evaluating the optic nerve head only evaluates structural information alone. A potential area of improvement is to develop algorithms with a more ‘holistic’ evaluation of structural and functional information, as well as information on other glaucoma risk factors such as family history and age.

Furthermore, there is a lack of prospective clinical trials applying AI in real world settings. For example, in the technologies for breast cancer screening with mammography, these issues have been particularly problematic [55]. Our team is actively trialling several AI models in optometry and GP settings and hope to publish relevant findings on the efficiency (such as accuracy, speed and acceptability of glaucoma screening) and cost-effectiveness of AI incorporation in screening programmes. In addition, real world AI implementation is impeded by challenges such as image quality, legal risks, and regulatory issues, which have barely been systematically summarised. Poor image quality can yield a high amount of ungradable images, which can lead to false positives and high health care cost. Furthermore, there have been concerns that increased reliance on AI may lead to deskilling of ophthalmic clinicians, and around privacy and cyber security as the large amount of personal information contained in the AI systems can increase the risk of data leakage. A systematic review on the patient privacy perspective on health information exchange [92] has found many patients express concerns about their health data privacy. Evidence-based protocols should be in place to safeguard algorithms and datasets against attacks. The introduction of AI-based technology may increase the cost of care, and if these costs are borne by individual patients, people of lower social economic status may remain undiagnosed. The best way for these tests to become widely available is for them to be included in health screening programmes promoted by national or private insurance companies. Our ongoing research, currently under review, pertains to cost-effectiveness analyses of AI-assisted glaucoma screening models within the context of Australia. The findings from our study indicate that the integration of AI assistance into population-based glaucoma screening programmes is cost-effective compared to traditional screening by optometrists (unpublished data, C. Jan 2023). These results suggest the economic feasibility of policy makers considering the adoption of universal glaucoma screening initiatives throughout Australia. However, it is imperative to note that the precise modalities and logistics for implementing such a programme remain to be defined. Furthermore, the medico-legal ramifications associated with placing reliance on technology for diagnostic purposes are expected to gain increasing significance and should be addressed in the context of healthcare policy and practice.

External validation studies are required to ensure the validity of deep learning algorithms and to better understand the mechanism underlying the technology or “thought process” of AI. Unlike human clinicians, current AI programmes are unable to take a holistic approach to patient care or consider other external contributing factors (such as social and psychological aspects) to management. Clinical trials are required to compare care models from practices with and without AI in real world primary care settings. AI is a tool that assists, not replaces, human clinicians.

### AI-development beyond academia

Our review focuses on the prevalence of undiagnosed glaucoma in Australia and the crucial role played by primary healthcare providers in glaucoma care within the Australian healthcare system. While our primary emphasis is not on industrial advancements, it is important to acknowledge the growing prominence of AI-enabled glaucoma screening outside the academic sphere, necessitating an exploration of the latest industry developments. For instance, as of January 2023, Eyenuk (US) has achieved the noteworthy accomplishment of securing the first European Union MDR Certification for autonomous AI detection of glaucomatous optic nerve damage utilising coloured fundus photographs. Furthermore, Eyetelligence (Australia), Digital Diagnostics (US), RetinaLyze (Denmark), and Ophthalmic Sciences (Israel) are actively engaged in the development of AI-based products aimed at facilitating glaucoma screening through the analysis of fundus photographs.

## Conclusion

This review highlights the significant challenge of glaucoma underdiagnosis, which can be attributed to variations in optic nerve head assessment, under-performing VF testing, and time constraints, consistent with challenges faced by primary healthcare practitioners in general in Australia. AI has shown the potential to mitigate these problems by carrying out glaucoma assessment (at least partially) in a consistent manner. The emergence of AI technology offers a promising solution to these challenges by enabling a more consistent and objective diagnosis of glaucoma. This is in contrast to the current situation, which is often characterised by inconsistent work-up and interpretation. AI algorithms have demonstrated high accuracy in diagnosing glaucoma and can provide a rapid diagnosis, thereby reducing the risk of misdiagnosis and enabling earlier treatment. Integrating AI into the diagnostic process for glaucoma has the potential to revolutionise the field and improve patient outcomes.

In primary eye care settings, advanced imaging technologies such as fundus photography and OCT, automated visual field testing, electronic health records, and large digital datasets are becoming increasingly available. These technologies can facilitate translational AI research to improve the evidence-based and consistent identification of glaucoma. As optometrists and GPs remain the first point of contact for patients with eye problems in Australia and other countries, the integration of AI into primary eye care settings has the potential to significantly improve glaucoma diagnosis and management.

## Data Availability

As this is a literature review, no original raw data was used.
